# Daten zu Behinderung, Gesundheit und Teilhabe in Deutschland – ein Update

**DOI:** 10.1007/s00103-026-04266-y

**Published:** 2026-07-13

**Authors:** Franziska Prütz, Birte Hintzpeter, Laura Krause, Anke-Christine Saß

**Affiliations:** Abteilung für Epidemiologie und Gesundheitsmonitoring, Robert Koch-Institut, Gerichtstraße 27, 13347 Berlin, Deutschland

**Keywords:** Behinderung, Gesundheit, Teilhabe, Deutschland, Surveys, Disability, Health, Participation, Germany, Surveys

## Abstract

Um politische Handlungsbedarfe zur Gesundheit und Teilhabe von Menschen mit Behinderungen ableiten zu können, werden belastbare, bevölkerungsbezogene Daten benötigt – auch im Sinne der UN-Behindertenrechtskonvention. Der Artikel enthält die Aktualisierung einer 2016 publizierten Übersicht zu entsprechenden Datenquellen in Deutschland.

Recherchiert wurden bevölkerungsbezogene Studien und Surveys, Daten der amtlichen Statistik und Prozessdaten ab 2015. Die Datenquellen sollten für die Gesundheitsberichterstattung (GBE) geeignet sein – insbesondere mit Blick auf Bevölkerungsbezug, Regelmäßigkeit der Erhebung und Verfügbarkeit – und den Empfehlungen der Vorstudie für den Teilhabesurvey entsprechen. Zur Kategorisierung wurde das dort beschriebene Stufenkonzept genutzt.

Identifiziert wurden 12 Studien und Surveys, 5 Datenquellen der amtlichen Statistik und 2 Prozessdatenquellen, die Daten zu Gesundheit und Teilhabe von Menschen mit Behinderungen mit unterschiedlichen Schwerpunkten und in unterschiedlicher Breite bzw. Tiefe enthalten. Im Vergleich zur vorherigen Übersicht ist mit dem Teilhabesurvey eine zentrale Datenquelle hinzugekommen. Lücken bestehen insbesondere bei Datenquellen zu Kindern und Jugendlichen.

In Deutschland existiert eine Vielzahl regelmäßig erhobener Datenquellen zu Behinderung, Gesundheit und Teilhabe, deren Potenzial für Auswertungen und Berichterstattung noch nicht ausgeschöpft ist. Weiterhin ist es notwendig, die Teilnahme an Studien barrierearm zu gestalten. Sinnvoll wäre die Nutzung von Standardinstrumenten bzw. die Integration eines kurzen Instruments zur Teilhabe in die Surveys. Ein Kernindikatorenset für die GBE könnte dazu beitragen, die evidenzbasierte Identifikation von Handlungsbedarfen zu unterstützen.

## Abstract (en)

Reliable population-based data are needed to identify policy needs regarding the health and participation of people with disabilities that are also in line with the UN Convention on the Rights of Persons with Disabilities. This article contains an update of an overview of relevant data sources in Germany published in 2016.

Population-based studies/surveys, official statistics and process data from 2015 onwards were researched. The data sources had to be suitable for health reporting (Gesundheitsberichterstattung, GBE)—in particular, they had to be population-based, be collected regularly, be readily available and comply with the recommendations of the preliminary study for the participation survey. The tiered concept described there was used for categorisation.

Twelve studies/surveys, five official statistical data sources and two process data sources were identified that contain data on the health and participation of people with disabilities with different focuses and varying breadth and depth. Compared to the previous overview, the participation survey has been added as a central data source. There are gaps, particularly in data sources on children and young people.

In Germany, there is quite a large number of regularly collected data sources on disability, health and participation, the potential of which has not yet been fully exploited for analyses and reporting. Participation in studies should be barrier-free, and the use of standard instruments or the integration of a short instrument on participation into the surveys would be useful. A set of core indicators for GBE could support the evidence-based identification of areas where action is needed.

## Einleitung

Behinderung ist nach wie vor eine „vernachlässigte soziale Determinante der Gesundheit“ [1]. Zwar nimmt die Aufmerksamkeit für die gesundheitlichen Belange und die Gesundheitsversorgung von Menschen mit Behinderungen zu, jedoch existieren trotz hoher Public-Health-Relevanz nur wenige Daten [2, 3]. Verlässliche Daten werden z. B. für die Gesundheitsberichterstattung (GBE) benötigt: Aus der Beschreibung der gesundheitlichen Lage der Bevölkerung oder einzelner Bevölkerungsgruppen können Problemstellungen und politische Handlungsbedarfe abgeleitet werden [4]. Zudem ist die Sammlung „geeigneter Informationen, einschließlich statistischer Angaben und Forschungsdaten“ in Art. 31 der UN-Behindertenrechtskonvention (UN-BRK) verankert [5]. Behinderungen resultieren aus der Interaktion von Personen mit ihrer Umwelt bzw. in den Worten der UN-BRK aus der „Wechselwirkung zwischen Menschen mit Beeinträchtigungen und einstellungs- und umweltbedingten Barrieren …, die sie an der vollen, wirksamen und gleichberechtigten Teilhabe an der Gesellschaft hindern“ ([5], Präambel, e). Auch die deutsche Sozialgesetzgebung nimmt in ihrem Behinderungsbegriff auf diese Wechselwirkungen Bezug (§ 2 SGB IX).

Nach den Daten des Mikrozensus leben in Deutschland rund 10,9 % der Menschen mit einer amtlich anerkannten Behinderung [6]. Laut Schwerbehindertenstatistik hatten 2023 rund 7,9 Mio. Menschen in Deutschland eine amtlich anerkannte Schwerbehinderung; dies entspricht 9,3 % der Bevölkerung [7]. Etwa die Hälfte dieser Personen ist weiblich, mehr als die Hälfte ist von körperlichen Behinderungen betroffen, rund ein Fünftel von zerebralen Störungen, geistigen und/oder seelischen Behinderungen [7]. Zur differenzierten Beschreibung der gesundheitlichen Lage ist die amtliche Statistik jedoch nicht ausreichend. Um herauszufinden, welche Datenquellen darüber hinaus in Deutschland verfügbar sind und für Auswertungen genutzt werden können, wurde 2016 eine Recherche von Datenquellen zur Gesundheit und Teilhabe von Menschen mit Behinderungen durchgeführt, die im Bundesgesundheitsblatt veröffentlicht wurde [2]. Der Begriff Teilhabe spiegelt die tatsächlichen Lebenslagen von Menschen mit Behinderungen wider [8]. Gesundheit ist in dieser Perspektive eine der Lebenslagedimensionen bzw. Teilhabefelder. Die Gesundheit einer Person hat Einfluss auf ihre Lebensqualität und ihre Teilhabechancen; somit ist Teilhabe zugleich eine wichtige Ressource für Lebensqualität und Gesundheit [8].

Als Anforderungen an Datenquellen wurden in der Recherche von 2016 eine Eignung für die GBE sowie die Empfehlungen aus der „Vorstudie für eine Repräsentativbefragung zur Teilhabe von Menschen mit Behinderung(en)“ [9] zugrunde gelegt. Kriterien für eine Eignung von Datenquellen für die GBE sind Bevölkerungsbezug, Regelmäßigkeit der Erhebungen, Verfügbarkeit sowie eine dauerhafte Etablierung in der deutschen Datenlandschaft [10, 11]. Die Empfehlungen aus der Vorstudie [9] sind sowohl auf einen eigenständigen Teilhabesurvey als auch auf die Integration entsprechender Fragestellungen in bestehende Surveysysteme anwendbar. Sie formulieren als allgemeine Grundsätze die Partizipation von Menschen mit Behinderungen und entsprechenden Institutionen bzw. Akteur:innen an der Vorbereitung, Durchführung und Auswertung der Datenerhebung sowie als Grundhaltung eine Ressourcen- statt Defizitorientierung. Zudem sollen verschiedene Lebensbereiche (Teilhabefelder) berücksichtigt werden, wie Familie und soziales Netz, Bildung und Ausbildung, Erwerbstätigkeit und materielle Lebenssituation, alltägliche Lebensführung, Gesundheit, Freizeit, Kultur und Sport, Sicherheit und Schutz der Person, politische und gesellschaftliche Teilhabe (vgl. dazu auch die Teilhabeberichte der Bundesregierung [8, 12, 13]). Als Konzept, um bestehende Befragungen um Ergänzungen zu Behinderungs- und Teilhabeaspekten zu erweitern, werden in den Empfehlungen 3 Stufen definiert [9], die in der Datenquellen-Recherche als Analysekategorien genutzt wurden. Diese umfassen:

- die Erhebung dauerhafter gesundheitlicher Beeinträchtigungen, die zu Einschränkungen im Alltagsleben führen, möglichst ergänzt durch Fragen nach amtlich anerkannter Behinderung und Alter bei Eintritt der Behinderung (1. Stufe),
- zusätzlich die Erfassung von Funktionsbeeinträchtigungen (körperlich, motorisch, sensorisch, kognitiv, psychisch), möglichst ergänzt durch Informationen zu chronischen Krankheiten und Schmerzen (2. Stufe),
- zusätzlich die Erfassung von Teilhabeeinschränkungen nach Lebensbereichen und durch die Umwelt (3. Stufe).

Da die Ergebnisse der Datenquellen-Recherche aus dem Bundesgesundheitsblatt [2] trotz abnehmender Aktualität weiterhin nachgefragt wurden, wird mit diesem Beitrag eine aktualisierte Fassung vorgelegt. Die ursprüngliche Recherche umfasste eine Internetrecherche, die Suche nach Datenquellen in zusammenfassenden Publikationen (Schnell und Stubbra [14], Schröttle et al. [9], Teilhabeberichterstattung [8, 12, 13]), im Informationssystem der GBE des Bundes (www.gbe-bund.de) sowie den akkreditierten Forschungsdatenzentren des Rats für Sozial- und Wirtschaftsdaten [2]. Die in dem Beitrag von 2016 gelisteten Studien wurden nachrecherchiert und die Angaben aktualisiert. Aus Gründen der Aktualität wurden nur Studien ab 2015 berücksichtigt, d. h., Studien, die älter waren, wurden aussortiert. Die Internetrecherche und die Recherche im Informationssystem der GBE des Bundes wurden wiederholt und zusätzlich ChatGPT für die Suche nach weiteren Datenquellen genutzt. Die Beschreibung der Datenquellen orientiert sich an den Verzeichnissen der Datenquellen in den Berichten „Gesundheit in Deutschland“ und „Gesundheitliche Lage der Frauen in Deutschland“ des RKI [3, 15] sowie am oben beschriebenen Stufenkonzept [9]. Ziel des Beitrags ist es, eine aktuelle Übersicht über Datenquellen zur Gesundheit und Teilhabe von Menschen mit Behinderungen in Deutschland zur Verfügung zu stellen.

## Ergebnisse: Datenquellen und Kategorisierung

### Beschreibung der Datenquellen

Die aktuelle Recherche ergab als Datenquellen für Informationen zu Behinderung und Teilhabe in Deutschland 12 Studien und Surveys sowie 5 Datenquellen aus der amtlichen Statistik. Weitere Datenquellen können der Kategorie Prozessdatenquellen und -berichte zugeordnet werden (Tab. 1). Daten zur erwachsenen Bevölkerung in unterschiedlichen Altersgruppen werden bzw. wurden in 7 Studien und Surveys erhoben: der „Allgemeinen Bevölkerungsumfrage der Sozialwissenschaften“ (ALLBUS), dem „European Social Survey“ (ESS), der Studie „Gesundheit in Deutschland aktuell“ (GEDA), dem RKI-Panel „Gesundheit in Deutschland“, dem Panel „Arbeitsmarkt und Soziale Sicherheit“ (PASS), der Studierendenbefragung und der Repräsentativbefragung zur Teilhabe von Menschen mit Behinderungen (Teilhabesurvey). In zwei Studien werden bzw. wurden Daten zu Kindern und Jugendlichen erhoben: „Aufwachsen in Deutschland: Alltagswelten“ (AID:A) sowie „Aufwachsen und Alltagserfahrungen von Jugendlichen mit Behinderung“; die Studie „Aufwachsen und Alltagserfahrungen von Jugendlichen mit Behinderung“ wurde trotz der fehlenden Periodizität aufgenommen, weil sie die zu dieser Altersgruppe vorhandenen Studien um wichtige Aspekte ergänzt. Zwei der gefundenen Studien fokussieren auf ältere Menschen: der „Deutsche Alterssurvey“ (DEAS) und der „Survey of Health, Aging and Retirement in Europe“ (SHARE). Zwei Studien, das Nationale Bildungspanel (NEPS) und das Sozio-oekonomische Panel (SOEP), erheben Daten über die gesamte Lebensspanne. Bei diesen sowie beim RKI-Panel handelt es sich zudem um Längsschnittstudien, sodass auch Entwicklungen im individuellen Lebensverlauf abgebildet werden können.

**Tab. 1:** Datenquellen zu Behinderung und Teilhabe (Darstellung in Anlehnung an [15])

Datenquelle	Beschreibung
*Studien/Surveys*	
*AID:A* („Aufwachsen in Deutschland: Alltagswelten“) Datenhalter: Deutsches Jugendinstitut (DJI)	AID:A erhebt Daten zum Aufwachsen von Kindern und Jugendlichen sowie zu den Lebenslagen von Erwachsenen und Familien in Deutschland. Ziel der Studie ist es, Faktoren und Konstellationen zu beschreiben, die den Lebenslauf und das Wohlergehen der Menschen prägen und beeinflussen. Dabei werden persönliche Einstellungen, Erfahrungen, Aktivitäten und Teilhabe ebenso wie sich wandelnde gesellschaftliche Rahmenbedingungen betrachtet. Besondere Berücksichtigung findet dabei die Einbindung in soziale Kontexte wie Familie, Freundschaften oder Peer-Groups. AID:A wird seit 2009 in regelmäßigen Abständen durchgeführt. Mit AID:A 2019 startete ein neuer Längsschnitt, der in regelmäßigen Abständen Informationen über Personen im Alter von 0 bis 32 Jahren erhebt. Die letzte Erhebung erfolgte 2023 (www.dji.de/aida/gesamtbeschreibung)
*ALLBUS* („Allgemeine Bevölkerungsumfrage der Sozialwissenschaften“) Datenhalter: GESIS Leibniz Institut für Sozialwissenschaften	In der Studienreihe ALLBUS werden seit 1980 alle 2 Jahre Zufallsstichproben der Bevölkerung mit einem teils konstanten, teils variablen Erhebungsprogramm befragt. Inhaltlich fokussiert ALLBUS auf wichtige sozialwissenschaftliche Themen, z. B. Einstellungen, Werte und Normen, Familie und Partnerschaft, Arbeit und Beruf, soziale Beziehungen, Religion u. v. m. Mit den ALLBUS-Daten lassen sich Entwicklungen und Wandel in der deutschen Gesellschaft in über 4 Jahrzehnten nachvollziehen. Pro Welle werden seit 1992 etwa 3500 Personen (erwachsene Wohnbevölkerung in Deutschland) befragt (www.gesis.org/allbus)
*Aufwachsen und Alltagserfahrungen von Jugendlichen mit Behinderung* Datenhalter: Deutsches Jugendinstitut (DJI)	Die Studie „Aufwachsen von Jugendlichen mit Behinderung“ (2018–2022) richtet den Blick auf die alterstypischen Belange und persönlichen Erfahrungen von Jugendlichen mit Behinderung in ihren Alltagswelten. Themenfelder der Studie sind: Freizeit, Freundschaften, Autonomie sowie Zufriedenheit und subjektives Erleben. Zielgruppe sind Jugendliche im Alter von 13 bis 18 Jahren mit einem festgestellten sonderpädagogischen Förderbedarf (SPF) in einem bzw. in einer Kombination der Bereiche Sehen, Hören, Sprache, Lernen, körperliche und motorische Entwicklung, emotionale und soziale Entwicklung und geistige Entwicklung. Die Erhebungsmethoden und -instrumente werden an die Möglichkeiten der teilnehmenden Jugendlichen angepasst. 2700 Teilnehmer:innen wurden rekrutiert (www.dji.de/Aufwachsen_mit_Behinderung)
*Deutscher Alterssurvey *(DEAS) Datenhalter: Deutsches Zentrum für Altersfragen (DZA)	Bundesweit repräsentative Quer- und Längsschnittbefragung von Personen, die 40 Jahre und älter sind. Im DEAS wird die Lebenssituation erfragt, u. a. beruflicher Status/Ruhestand, gesellschaftliche Partizipation und nachberufliche Aktivitäten, familiäre und sonstige soziale Kontakte, wirtschaftliche Lage, Gesundheit und Wohlbefinden. Die erste Befragung wurde im Jahr 1996 durchgeführt, die letzte im Jahr 2023. An dieser Welle, einer reinen Wiederholungsbefragung, nahmen rund 5000 Personen teil. Die Daten dienen als Informationsquelle sowohl für die sozial- und verhaltenswissenschaftliche Forschung als auch für die Sozialberichterstattung (www.dza.de/forschung/deas)
*ESS* („European Social Survey“) Datenhalter: ESS ERIC Headquarter	Internationaler Survey mit Beteiligung der europäischen Mitgliedsstaaten sowie Albanien, Großbritannien, Island, Israel, Kosovo, Norwegen, Russische Föderation, Türkei und Ukraine. Er wird seit 2001 alle 2 Jahre durchgeführt. Im Erhebungsjahr 2023/2024 nahmen 31 Länder teil. Es wird jeweils eine neue Stichprobe gezogen; Grundgesamtheit ist die Wohnbevölkerung im Alter ab 15 Jahren. Der Survey untersucht Einstellungen, Meinungen und Verhaltensmuster in den teilnehmenden Ländern. Neben einem Kernmodul zu politischen Einstellungen, Vertrauen, subjektivem Wohlbefinden sowie soziodemografischen Angaben werden die einzelnen Wellen durch rotierende Module ergänzt (www.europeansocialsurvey.org)
*GEDA bzw. GEDA-EHIS* („Gesundheit in Deutschland aktuell“) und *Panel „Gesundheit in Deutschland*“ Datenhalter: Robert Koch-Institut (RKI)	GEDA ist eine repräsentative Gesundheitsbefragung von Personen im Alter ab 18 Jahren (in der Regel), die im Rahmen des bundesweiten Gesundheitsmonitorings des RKI von 2009 bis 2024 regelmäßig durchgeführt wurde. Pro Survey wurden mehr als 20.000 Teilnehmende zu (chronischen) Krankheiten, dem Gesundheitsverhalten, der Inanspruchnahme von Leistungen des Gesundheitssystems und soziodemografischen Merkmalen befragt (www.rki.de/geda). Im Jahr 2024 gab es eine Umstellung auf das RKI-Panel „Gesundheit in Deutschland“ (www.rki.de/panel). In die GEDA-Wellen 2014/2015-EHIS, 2019/2020-EHIS und RKI-Panel 2025-EHIS wurde das Instrument der Europäischen Gesundheitsbefragung (EHIS) integriert
*NEPS* („Nationales Bildungspanel“) Datenhalter: Leibniz-Institut für Bildungsverläufe e. V. (LIfBi) an der Otto-Friedrich-Universität Bamberg	Ziel des NEPS ist es, Längsschnittdaten zu Kompetenzentwicklungen und Bildungsverläufen in formalen, nichtformalen und informellen Kontexten über die gesamte Lebensspanne zu erheben. Dazu ist es notwendig, dass Kompetenzentwicklungen nicht nur im Kindergarten oder im allgemeinbildenden Schulsystem, sondern auch in der beruflichen Ausbildung, im Studium und nach Verlassen des Bildungssystems gemessen werden. Um dies zu erreichen, wurden seit 2009 7 Startkohorten mit insgesamt mehr als 70.000 Personen begleitet. Die Teilnehmenden werden über einen längeren Zeitraum regelmäßig befragt. Zusätzlich werden etwa 50.000 Personen aus deren Umfeld, wie Eltern und pädagogisches Fachpersonal, befragt (www.neps-data.de)
*PASS* („Panel Arbeitsmarkt und soziale Sicherung“) Datenhalter: Institut für Arbeitsmarkt und Berufsforschung (IAB)	Jährlich stattfindende Haushaltsbefragung. Die von PASS erhobenen Daten geben Aufschluss über die Dynamik des Grundsicherungsbezugs und die soziale Lage der sich darin befindenden Haushalte. Die Befragung wird seit 2006 jährlich durchgeführt, mit insgesamt über 42.000 Teilnehmenden zwischen 15 und 65 Jahren. In ca. 10.000 Haushalten werden jährlich rund 16.000 Personen befragt. Über den Kernbereich „Beschäftigung und Arbeitslosigkeit“ hinaus bietet das PASS ein breites Fragenspektrum, das z. B. auch zahlreiche soziodemografische Merkmale oder subjektive Indikatoren (wie Zufriedenheit, Ängste und Sorgen, Erwerbsorientierung) beinhaltet (https://iab.de/das-iab/befragungen/iab-haushaltspanel-pass)
*SHARE* („Survey of Health, Aging and Retirement in Europe“) Datenhalter: SHARE Research Data Center	Multidisziplinäre, internationale Langzeit-Panelstudie zur Erforschung der sozialen, wirtschaftlichen und gesundheitlichen Situation alternder Menschen in Europa. SHARE erhebt seit 2004 alle 2 Jahre Befragungsdaten der Altersgruppe 50 Jahre und älter in 28 europäischen Ländern (und Israel) und führt in Untergruppen zusätzlich Untersuchungen durch. Die 9. Welle der Untersuchung fand von 2021 bis 2022 statt. Insgesamt wurden rund 164.000 Menschen in mehr als 616.000 Interviews befragt (https://share-eric.eu)
*SOEP* („Sozio-oekonomisches Panel“) Datenhalter: Deutsches Institut für Wirtschaftsforschung (DIW)	Repräsentative Befragung privater Haushalte, die seit 1984 jährlich im Paneldesign durchgeführt wird. Zurzeit werden jedes Jahr in Deutschland etwa 30.000 Teilnehmende in fast 11.000 Haushalten befragt (alle Altersgruppen). Die Daten geben Auskunft zu Einkommen, Erwerbstätigkeit, Bildung und enthalten Fragen zur Gesundheit. Aufgrund des Paneldesigns können langfristige soziale und gesellschaftliche Trends verfolgt werden (www.diw.de/soep)
*Studierendenbefragung in Deutschland* Datenhalter: Deutsches Zentrum für Hochschul- und Wissenschaftsforschung	Die „Studierendenbefragung in Deutschland“ ist eine bundesweite, repräsentative Befragung von Studierenden an deutschen Hochschulen. Sie erhebt Daten zur Lebens‑, Studien-, sozialen und wirtschaftlichen Situation der Studierenden und wird alle 4 Jahre durchgeführt. Seit 2021 gibt es diese Studie als Zusammenführung der Sozialerhebung des Deutschen Studentenwerks, des Studierendensurveys und der Befragung „beeinträchtigt studieren“ (best). In der ersten Welle im Jahr 2021 wurden 188.000 Studierende von 250 Hochschulen befragt. Die aktuelle Befragung wurde im ersten Halbjahr 2025 durchgeführt, über eine Million Studierende waren zur Teilnahme eingeladen (https://die-studierendenbefragung.de)
*Teilhabesurvey *(„Repräsentativbefragung zur Teilhabe von Menschen mit Behinderungen“) Datenhalter: Forschungsdatenzentrum der Bundesanstalt für Arbeitsschutz und Arbeitsmedizin (FDZ-BAuA)	Repräsentativbefragung zur Teilhabe von Menschen mit Behinderungen und Beeinträchtigungen. Der Teilhabesurvey befragt sowohl Personen in Privathaushalten als auch Bewohnerinnen und Bewohner in besonderen Wohnformen, im betreuten Wohnen, in Alten- und Pflegeeinrichtungen sowie wohnungslose Menschen. In der 1. Welle (2017–2021) wurden rund 25.000 Personen, in der 2. Welle (2023–2024) rund 14.000 Personen mit und ohne Beeinträchtigungen und Behinderungen zu ihrer Teilhabe in allen wichtigen Lebensbereichen befragt (www.baua.de/DE/Forschung/Forschungsdaten/Teilhabesurvey-BMAS)
*Amtliche Statistik*	
*EU-LFS *(„Labour Force Survey“) Datenhalter: Statistisches Amt der Europäischen Union (Eurostat)	Statistik zur Arbeitsmarktbeteiligung der Bevölkerung ab 14 Jahren, die in allen Staaten der EU jährlich in harmonisierter Form durchgeführt wird. In Deutschland sind die Fragen in den Mikrozensus integriert. Seit 1999 werden zusätzlich Ad-hoc-Module mit wechselnden Themenschwerpunkten durchgeführt. Daten zu Behinderung und Teilhabe finden sich in den 2002 und 2011 durchgeführten Modulen zur Beschäftigung von Menschen mit Behinderung. Mit der Neuregelung der EU-Sozialstatistiken werden seit 2021 regelmäßige Module zu Behinderung und Teilhabe am Arbeitsmarkt durchgeführt. So ist etwa alle 2 Jahre eine Messung von Behinderung im EU-LFS integriert, die auf dem Global Activity Limitation Indicator (GALI) basiert (https://ec.europa.eu/eurostat/web/lfs)
*EU-SILC *(„EU Statistics on Income and Living Conditions“) Datenhalter bis 2019: Statistisches Amt der Europäischen Union (Eurostat); Datenhalter seit 2020: Statistisches Bundesamt	EU-SILC ist eine jährliche repräsentative Erhebung in den EU-Mitgliedstaaten. Zwischen 2005 und 2019 wurde der deutsche Teil („Leben in Europa“) als eigenständige Erhebung durchgeführt und jedes Jahr rund 14.000 Haushalte schriftlich befragt (Haushaltsfragebogen und Personenfragebogen für jedes Haushaltsmitglied ab 16 Jahren). Seit 2020 ist die Erhebung als Unterstichprobe in den Mikrozensus integriert. Dies ermöglicht eine größere und repräsentativere Stichprobe für Deutschland (Befragung von rund 40.000 Haushalten jährlich). Da die Haushalte in 4 aufeinanderfolgenden Jahren befragt werden, sind auch Längsschnittauswertungen möglich. Ein Vergleich mit den Ergebnissen bis 2019 ist allerdings nicht möglich. Inhaltliche Schwerpunkte der Befragung sind Einkommen, Armut und Lebensbedingungen. Erhoben werden dabei u. a. Informationen zu Wohnsituation, materieller Entbehrung, sozialer Teilhabe und Gesundheit (www.destatis.de)
*Mikrozensus * Datenhalter: Statistisches Bundesamt	Seit 1957 durchgeführte, repräsentative Haushaltsbefragung der amtlichen Statistik in Deutschland. Rund 830.000 zufällig ausgewählte Personen (1 % der Bevölkerung) werden jährlich befragt. Die Teilnahme ist verpflichtend. Inhaltliche Schwerpunkte: Erwerbstätigkeit, Aus- und Weiterbildung, Einkommen, Angaben zur Person (u. a. Migrationshintergrund). Alle 4 Jahre werden Fragen zur Gesundheit gestellt (Teilnahme freiwillig), amtlich anerkannte Behinderung und der Grad der Behinderung werden seit 2017 jährlich erhoben (www.destatis.de)
*Statistik der allgemeinbildenden Schulen * Datenhalter: Statistisches Bundesamt	Die Schulstatistik stellt die Entwicklung des Schulwesens dar und weist für jedes Schuljahr u. a. die Anzahl der Schülerinnen und Schüler mit sonderpädagogischer Förderung nach Schularten, Bildungsbereichen und Förderschwerpunkten aus. Die bis 2023 jährlich und seitdem alle 2 Jahre erscheinenden Bildungsberichte bereiten diese und andere Daten (z. B. aus der Kinder- und Jugendhilfestatistik, s. unten) auf und vermitteln ein Gesamtbild der Inklusion im Bildungsbereich (www.destatis.de)
*Statistik der Kinder- und Jugendhilfe* Datenhalter: Statistisches Bundesamt	Die Statistiken der Kinder- und Jugendhilfe umfassen Angaben zu einem breiten Spektrum der im Kinder- und Jugendhilfegesetz (SGB VIII) geregelten Aufgaben der Jugendämter. Darunter sind auch Daten zu Kindern mit einrichtungsgebundener Eingliederungshilfe in Tageseinrichtungen und in öffentlich geförderter Kindertagespflege (darunter auch Tageseinrichtungen mit integrativer Betreuung und Tageseinrichtungen für Kinder mit Behinderung). Die Statistik wurde 2022 neu konzipiert (Trägerstatistik) und wird nicht mehr jährlich, sondern alle 2 Jahre durchgeführt. Ein Vergleich der Ergebnisse ist nur eingeschränkt möglich. Die Daten werden auch für die Bildungsberichterstattung genutzt (www.destatis.de)
*Statistik der schwerbehinderten Menschen* Datenhalter: Statistisches Bundesamt	Seit 1985 wird alle 2 Jahre eine Bundesstatistik über schwerbehinderte Menschen durchgeführt. Diese umfasst die Zahl der schwerbehinderten Menschen mit gültigem Ausweis, persönliche Merkmale (Alter, Geschlecht, Staatsangehörigkeit, Wohnort) sowie Art, Ursache und Grad der Behinderung. Die Daten stammen von den Versorgungsämtern, Landesversorgungsämtern und versorgungsärztlichen Untersuchungsstellen (www.destatis.de)
*Prozessdaten und -berichte*	
*Statistik der Deutschen Rentenversicherung/Reha-Berichte* Datenhalter: Deutsche Rentenversicherung (DRV)	Die Statistik der Deutschen Rentenversicherung umfasst neben Angaben zu aktiv Versicherten, Rentnerinnen und Rentnern, Rentenneuanträgen und Rentenzugängen in der gesetzlichen Rentenversicherung auch Renten wegen verminderter Erwerbsfähigkeit und von der gesetzlichen Rentenversicherung getragene Leistungen zur Rehabilitation und Teilhabe. In den jährlich erscheinenden Reha-Berichten befinden sich die wichtigsten aktuellen Daten und Fakten zur medizinischen und beruflichen Rehabilitation der Rentenversicherung (https://statistik-rente.de/drv/extern)
*Statistik der Bundesagentur für Arbeit * Datenhalter: Bundesagentur für Arbeit (BA)	Mehrere regelmäßig erhobene Statistiken der BA enthalten Angaben zu Behinderung und Teilhabe: Beschäftigungsstatistik und Arbeitslosenstatistik zeigen die Arbeitsmarktbeteiligung von Menschen mit Schwerbehinderung oder einer der Schwerbehinderung gleichgestellten Beeinträchtigung. Die Statistik zur Teilhabe behinderter Menschen am Arbeitsleben (Reha-Statistik) berichtet über Rehabilitanden, für die die BA als Rehabilitationsträger zuständig ist (https://statistik.arbeitsagentur.de) [13]

Die Datenquellen aus der amtlichen Statistik umfassen sowohl Haushaltsbefragungen (Mikrozensus, EU Statistics on Income and Living Conditions (EU-SILC)) als auch Daten aus dem Sozial- und Bildungswesen (Schule, Kinder- und Jugendhilfe, Versorgungsämter).

Prozessdaten der Deutschen Rentenversicherung (DRV) und der Bundesagentur für Arbeit (BA) können ebenfalls hilfreich sein. EU-SILC ist seit 2020 als Unterstichprobe in den Mikrozensus integriert. Dies führt nicht nur zu einer größeren Stichprobe, sondern aufgrund der Teilnahmepflicht am Mikrozensus auch zu einer höheren Repräsentativität für die Bevölkerung [16]. Allerdings ergibt sich daraus auch ein Zeitreihenbruch. Der Mikrozensus selbst wurde 2020 neu ausgerichtet. Aufgrund der zunehmenden Schwierigkeiten, den Anteil behinderter Menschen in Einrichtungen zu schätzen, erfolgt seit 2022 kein Abgleich der Daten mehr mit der Schwerbehindertenstatistik. Damit sind die Zahlen deutlich niedriger als in den Vorjahren und als Untergrenzen zu interpretieren [6].

### Kategorisierung der Datenquellen

Die Kategorisierung der Datenquellen nach dem Stufenkonzept wird in Tab. 2 dargestellt. Mit Blick auf Stufe 1 (Vorliegen dauerhafter gesundheitlicher Beeinträchtigungen, die zu Einschränkungen im Alltagsleben führen, möglichst erweitert um die Frage nach amtlich festgestellter Schwerbehinderung und dem Alter bei Eintritt der Behinderung) zeigt sich, dass in den meisten der aufgeführten Datenquellen Angaben zum Vorliegen einer amtlich anerkannten Behinderung vorhanden sind. DEAS, NEPS, PASS und SOEP weisen zusätzlich das Datum der amtlichen Anerkennung aus. GEDA, das RKI-Panel, PASS, SOEP und SHARE nutzen den globalen Indikator für Aktivitätseinschränkungen (Global Activity Limitation Indicator, GALI) zur Erhebung von Behinderung, der den Schweregrad einer lang andauernden Beeinträchtigung üblicher Alltagsaktivitäten aufgrund gesundheitlicher Probleme beschreibt [17]. Das Instrument wird auch in den von Eurostat koordinierten sozialstatistischen Stichprobenerhebungen in der EU wie EU-SILC verwendet [17].

**Tab. 2:** Kategorisierung der Datenquellen zu Behinderung und Teilhabe (ab 2015, Stufenkonzept basierend auf [9], Abkürzungen s. Tab. 1)

Datenquellen	Stufe 1	Stufe 2	Stufe 3
	Einschränkungen im Alltagsleben durch das Vorliegen dauerhafter gesundheitlicher Beeinträchtigungen	Zusätzlich: Funktionsbeeinträchtigungen, möglichst ergänzt um chronische Krankheiten und Schmerzen	Zusätzlich: Teilhabeeinschränkungen nach Lebensbereichen und durch die Umwelt
*Studien/Surveys*			
*AID:A (2023)*	Behinderungen, physische oder psychische Beeinträchtigungen	–	Soziale Eingebundenheit, aktive Partizipation in sozialen Gruppen (z. B. ehrenamtliches Engagement, Freizeitaktivitäten) sowie Nutzung kultureller und sozialstaatlicher Angebote (Auswertungen zur Teilhabe durch Verknüpfung mit Gesundheitsvariablen möglich)
*ALLBUS*	Beeinträchtigung alltäglicher Aktivitäten aufgrund körperlicher, seelischer oder emotionaler gesundheitlicher Probleme; amtlich festgestellte Schwerbehinderung oder Erwerbsminderung; Grad der Behinderung	Einzelne körperliche Funktionsbeeinträchtigungen (Treppen steigen, Schweres heben)	Mitgliedschaft in Organisationen oder Vereinen, Freizeitaktivitäten, soziale Netzwerke
*Aufwachsen und Alltagserfahrungen von Jugendlichen mit Behinderung*	Vorhandensein Schwerbehindertenausweis (einbezogen wurden nur Jugendliche mit einem festgestellten sonderpädagogischen Förderbedarf (SPF))	Nutzung von Rollstuhl/Rollator, Sehhilfen/Blindenstock, Hörhilfen/Gebärdensprache	Freizeitaktivitäten, Vereine, Jugendgruppen, Beziehungen zu Gleichaltrigen, Partnerschaft, Nutzung digitaler Medien, Selbstbestimmung im Alltag, Eigenständigkeit bei Mobilität, Barrieren der persönlichen Zukunftsplanung
*Deutscher Alterssurvey (2023)*	Vorübergehende oder dauerhafte Einschränkung der eigenen Gesundheit durch schwere Krankheit oder Unfall; Vorliegen einer amtlich festgestellten Behinderung; Grad der amtlich festgestellten Behinderung; Jahr der erstmaligen Anerkennung der amtlich festgestellten Behinderung	Sehprobleme, Hörprobleme; Einschränkung in der Ausübung verschiedener Alltagsaktivitäten (Liste) aufgrund des Gesundheitszustands	Soziale Netzwerke und Unterstützung, Freizeitaktivitäten, bürgerschaftliches/ehrenamtliches Engagement, gesellschaftliche Partizipation etc. (Auswertungen zur Teilhabe durch Verknüpfung mit Gesundheitsvariablen möglich)
*Studierendenbefragung in Deutschland*	Amtlich anerkannte Behinderung	Einschränkungen beim Hören, Sehen, Bewegung, psychische Erkrankung, chronische Erkrankung u. a.	Auswirkungen gesundheitlicher Beeinträchtigungen und Herausforderungen im Studienalltag (z. B. Teilnahme an Lehrveranstaltungen, Prüfungen, Mobilität, digitale Zugänge), Fragen zu Unterstützungsleistungen
*ESS (2023/2024)*	Beeinträchtigung alltäglicher Aktivitäten durch Krankheit, seelische Krankheit oder Behinderung	Einschränkungen im Alltag bezogen auf einzelne gesundheitliche Probleme (Liste von Krankheiten und Beschwerden)	Diskriminierungserfahrungen (z. B. aufgrund von Behinderung), politische Partizipation, soziale Netzwerke
*GEDA (2014/2015-EHIS)*	Beeinträchtigung alltäglicher Aktivitäten aus gesundheitlichen Gründen (GALI-Frage); amtlich anerkannte Behinderung; Grad der Behinderung	Körperliche und sensorische Funktionseinschränkungen (Schwierigkeiten beim Sehen, Hören, Gehen oder Treppensteigen); Schmerzen; Einschränkungen in instrumentellen Aktivitäten des täglichen Lebens (iADL), z. B. Telefonieren, Einkaufen, Erledigen von Bankgeschäften, Haushaltsführung, Einnahme von Medikamenten und Nutzung von Transportmitteln	–
*GEDA (2019/2020-EHIS)*	Beeinträchtigung alltäglicher Aktivitäten aus gesundheitlichen Gründen (GALI-Frage)	Einschränkungen in instrumentellen Aktivitäten des täglichen Lebens (iADL), z. B. Telefonieren, Einkaufen, Erledigen von Bankgeschäften, Haushaltsführung, Einnahme von Medikamenten und Nutzung von Transportmitteln: Einschränkungen bei grundlegenden Tätigkeiten der Erfüllung der Grundbedürfnisse (ADL), z. B. Essen, Körperhygiene, Aufstehen, Ankleiden oder Toilettenbenutzung	–
*GEDA 2022 und GEDA 2023*	Beeinträchtigung alltäglicher Aktivitäten aus gesundheitlichen Gründen (GALI-Frage)	–	–
*RKI-Panel „Gesundheit in Deutschland“, Panel 2025-EHIS*	Beeinträchtigung alltäglicher Aktivitäten aus gesundheitlichen Gründen (GALI-Frage); amtlich anerkannte Behinderung; Grad der Behinderung	Körperliche und sensorische Funktionseinschränkungen (Schwierigkeiten beim Sehen, Hören, Gehen oder Treppensteigen); Einschränkungen bei Aktivitäten des täglichen Lebens (ADL, iADL)	Teilhabebarrieren in verschiedenen Lebensbereichen: Mobilität, Transport, Zugang zu öffentlichen Gebäuden, soziale Aktivitäten, Internetnutzung
*NEPS* (Startkohorten)	Amtlich anerkannte Behinderung; Anerkennungsjahr der Behinderung; Grad der Behinderung	Art der Behinderung (offene Angabe) Förderschwerpunkt: Sehen, Lernen, Autismus, Sprache, geistige Entwicklung, körperliche und motorische Entwicklung, Hören, emotionale und soziale Entwicklung	Politische Teilhabe (Beteiligung); Teilhabe in Vereinen, Ehrenamt, soziale Organisationen
*PASS* (aktuell: Welle 18)	Vorliegen einer amtlich festgestellten Behinderung; Grad der amtlich festgestellten Behinderung; Datum der Anerkennung der amtlich festgestellten Behinderung; Einschränkungen in der Aufnahme einer Erwerbstätigkeit aufgrund des Gesundheitszustands	Schwerwiegende gesundheitliche Beeinträchtigungen (körperliche Behinderung oder Einschränkung, Seh- oder Hörbehinderung, Anfallsleiden, organische Erkrankungen, psychische oder seelische Erkrankung oder Behinderung), Art der Behinderung bzw. gesundheitlichen Einschränkung, z. B. Seh- oder Hörbehinderung, Krebserkrankung	Soziale Teilhabe; Nichtteilhabe an Freizeitaktivitäten; Aktivitäten in Organisationen oder Vereinen, Netzwerke, Stigmabewusstsein
*SHARE* (Welle 9)	Einschränkungen alltäglicher Verrichtungen aufgrund gesundheitlicher Probleme (GALI-Frage); langwierige Gesundheitsprobleme, Krankheiten oder Behinderungen; gesundheitliche Probleme oder Behinderungen, welche Art oder Umfang der Erwerbstätigkeiten einschränken	Seh- und Hörfähigkeit; Schwierigkeiten bei alltäglichen Verrichtungen (Liste zu funktionalen Einschränkungen); Skala zu instrumentellen Aktivitäten des täglichen Lebens; Tests zu kognitiven Fähigkeiten und körperlichen Funktionsfähigkeiten	Teilhabe an sozialen Aktivitäten und Zufriedenheit mit Teilhabe/Nichtteilhabe an aufgeführten Aktivitäten; Gefühle der Einsamkeit, Isolation, des am Rande Stehens; Vorhandensein baulicher Maßnahmen, um Menschen mit körperlichen Behinderungen zu helfen
*SOEP 2018* (SOEP-Innovations-Stichprobe)	Amtlich festgestellte Erwerbsminderung, amtlich anerkannte Behinderung; Grad der Erwerbsminderung bzw. Schwerbehinderung	Beeinträchtigungen beim Treppensteigen; Einschränkung in der Arbeit oder alltäglichen Beschäftigungen aufgrund gesundheitlicher Probleme	Einschränkung in sozialen Kontakten wegen gesundheitlicher oder seelischer Probleme
*SOEP 2019* (SOEP-IS 2019 – Fragebogen für die SOEP-Innovations-Stichprobe)	Amtlich festgestellte Erwerbsminderung, amtlich anerkannte Behinderung; Grad der Erwerbsminderung bzw. Schwerbehinderung; Zeitpunkt der amtlich festgestellten Erwerbsminderung bzw. Behinderung; Art der zugrunde liegenden Beeinträchtigung	–	Freizeitaktivitäten wie Essen gehen, Besuche von Nachbarn, Freunden oder Verwandten; Ausflüge; Beteiligung in Parteien, Vereinen oder Verbänden; Kinobesuche usw.
*SOEP 2020* (SOEP-Core Personenfragebogen 2020)	Amtlich festgestellte Erwerbsminderung, amtlich anerkannte Behinderung; Grad der Erwerbsminderung bzw. Schwerbehinderung; Zeitpunkt der amtlich festgestellten Erwerbsminderung bzw. Behinderung; Grund für die amtlich festgestellte Erwerbsminderung bzw. Schwerbehinderung; Bezug von Rente wegen teilweiser oder voller Erwerbsminderung	Beeinträchtigungen beim Treppensteigen; Einschränkung in der Arbeit oder alltäglichen Beschäftigungen aufgrund gesundheitlicher Probleme	Einschränkung in sozialen Kontakten wegen gesundheitlicher oder seelischer Probleme
*SOEP 2023* (SOEP-Core Personenfragebogen 2023)	Beeinträchtigung alltäglicher Aktivitäten aus gesundheitlichen Gründen (GALI-Frage); amtlich festgestellte Erwerbsminderung, amtlich anerkannte Behinderung; Grad der Erwerbsminderung bzw. Schwerbehinderung; Zeitpunkt der amtlich festgestellten Erwerbsminderung bzw. Behinderung; Grund für die amtlich festgestellte Erwerbsminderung bzw. Schwerbehinderung; Bezug von Rente wegen teilweiser oder voller Erwerbsminderung	–	–
*Teilhabesurvey Welle 1 (2019–2020)*	Dauerhafte Beeinträchtigung oder Behinderung; Alter bei Eintritt der Beeinträchtigung; am stärksten bei Alltagsaktivitäten einschränkende Beeinträchtigung; Ausmaß der Beeinträchtigung, amtlich anerkannte Behinderung; Grad der Behinderung; gesetzliche Betreuung; amtliche Feststellung einer Erwerbsminderung	Stärke der Beeinträchtigung; Unterstützung; Einschränkung der Alltagsaktivitäten; dauerhafte Beeinträchtigung oder Behinderung, z. B. beim Sehen, Hören, Sprechen, Lernen, Denken, Erinnern oder Orientieren im Alltag, durch Schmerzen, durch eine chronische Erkrankung usw.	Wohnsituation; Unterstützungsbedarf im täglichen Leben; aktueller Erwerbsstatus; subjektive Einschätzung der Teilhabe *Teilhabebereiche der UN-BRK*: Sicherung von Meinungsfreiheit, Teilhabe am Arbeitsleben; Förderung von Gesundheit und Prävention, Zugang zu Rehabilitation und Pflege; soziale Sicherung; unabhängige Lebensführung und gesellschaftliche, politische sowie kulturelle Teilhabe; Zugänglichkeit von Gebäuden und Infrastruktur usw.
*Teilhabesurvey Welle 2 (2023–2024)*	Dauerhafte Beeinträchtigung oder Behinderung; Alter bei Eintritt der Beeinträchtigung; am stärksten bei Alltagsaktivitäten einschränkende Beeinträchtigung; Ausmaß der Beeinträchtigung, amtlich anerkannte Behinderung; Grad der Behinderung; gesetzliche Betreuung; amtliche Feststellung einer Erwerbsminderung	Stärke der Beeinträchtigung; Unterstützung; Einschränkung der Alltagsaktivitäten; dauerhafte Beeinträchtigung oder Behinderung, z. B. beim Sehen, Hören, Sprechen, Lernen, Denken, Erinnern oder Orientieren im Alltag, durch Schmerzen, durch eine chronische Erkrankung usw.	Wohnsituation; Unterstützungsbedarf im täglichen Leben; aktueller Erwerbsstatus; subjektive Einschätzung der Teilhabe *Teilhabebereiche der UN-BRK: *Freizügigkeit; Zugang zu Bildung; retrospektive Betrachtung zu Beeinträchtigung und Gesundheit; Sicherung des Zugangs zu Ehe, Familienbildung, Partnerschaft, Sexualität; Sicherung der Freiheit, Schutz vor Gewalt, Schutz der Unversehrtheit und Sicherheit
*Amtliche Statistik*			
*Statistik der schwerbehinderten Menschen*	Amtlich anerkannte Schwerbehinderung, Grad der Behinderung	Ursache der Behinderung, Art der Behinderung	–
*Mikrozensus*	Amtlich anerkannte Behinderung, Grad der Behinderung in Privathaushalten	–	Schulabschluss, Berufsabschluss, Erwerbstätigkeit/-losigkeit, Stellung im Beruf, überwiegender Lebensunterhalt
*EU-SILC*	Allgemeiner Gesundheitszustand, lang andauernde chronische Krankheiten, krankheitsbedingte Einschränkungen bei alltäglichen Arbeiten (GALI-Frage)	Einschränkungen beim Sehen, Hören, Gehen, Treppensteigen, Erinnern oder Konzentrieren, Einschränkungen in instrumentellen Aktivitäten des täglichen Lebens	Einkommen, Armutsgefährdung, finanzielle Situation des Haushalts (Selbsteinschätzung), Wohnsituation, Bildungsabschluss, Erwerbsstatus, Verzicht auf Arztbesuch, Diskriminierungserfahrungen
*EU-LFS*	Allgemeiner Gesundheitszustand, lang andauernde chronische Krankheiten, krankheitsbedingte Einschränkungen bei alltäglichen Arbeiten (GALI-Frage)	Einschränkungen bei Alltagsaktivitäten	Erwerbstätigkeit, Erwerbslosigkeit, Behinderung am Arbeitsplatz, bedingt durch ein lang andauerndes Gesundheitsproblem oder eine Einschränkung in Alltagsaktivitäten, Unterstützung am Arbeitsplatz, Barrieren auf dem Arbeitsmarkt
*Statistik der Kinder- und Jugendhilfe*	Art der Behinderung, einrichtungsgebundene Eingliederungshilfe	–	Tageseinrichtung mit integrativer Betreuung, Tageseinrichtung für Kinder mit Behinderung, Art der in Anspruch genommenen Leistungen (z. B. Leistungen zur sozialen Teilhabe, Leistungen zur Teilhabe an Bildung, Leistungen zur Teilhabe am Arbeitsleben)
*Statistik zu allgemeinbildenden Schulen*	Sonderpädagogischer Förderbedarf	Förderschwerpunkte (z. B. Lernen, emotionale und soziale Entwicklung, Sprache, geistige Entwicklung, körperliche und motorische Entwicklung)	Schultyp (allgemeine Schule, Förderschule) Unterstützungsbedarf (Informationen zu den benötigten Ressourcen, z. B. Einsatz von Schulbegleiter:innen)
*Prozessdaten und -berichte*			
*Statistik der Deutschen Rentenversicherung, Reha-Berichte*	Bezug einer Erwerbsminderungsrente, Leistungen zur Rehabilitation	Funktionseinschränkungen (z. B. Einschränkungen beim Heben/Tragen, bei der Konzentrationsfähigkeit), Diagnosehauptgruppen	Leistungen zur beruflichen Rehabilitation/Teilhabe am Arbeitsleben (einschl. Leistungsspektrum; u. a. Leistungen zur beruflichen Bildung, einschl. Krankheitsspektrum)
*Statistik der Bundesagentur für Arbeit* Beschäftigungsstatistik Arbeitslosenstatistik Reha-Statistik Berichte zur Arbeitsmarktsituation von schwerbehinderten Menschen	Art und Grad der Behinderung, Amtlich anerkannte Schwerbehinderung oder eine der Schwerbehinderung gleichgestellte Behinderung	–	Beschäftigt nach Wirtschaftszweigen, Arbeitslosigkeit, Berufsausbildung bei Arbeitslosigkeit, Inanspruchnahme arbeitsmarktpolitischer (Förder‑)Maßnahmen, Maßnahmen zur beruflichen Rehabilitation

Angaben zur Stufe 2 (zusätzliche Erfassung von Funktionsbeeinträchtigungen (körperlich, motorisch, sensorisch, kognitiv, psychisch), möglichst ergänzt durch Informationen zu chronischen Krankheiten und Schmerzen) werden in den betrachteten Studien und Surveys sehr heterogen erfasst. Seh- und Hörprobleme sind häufig enthalten, dies gilt auch für Einschränkungen in der Ausübung verschiedener Alltagsaktivitäten. Angaben zu Schmerzen finden sich in GEDA, dem RKI-Panel, dem SOEP und dem Teilhabesurvey. Die Studie „Aufwachsen und Alltagserfahrungen von Jugendlichen mit Behinderung“ stellt Informationen zur Nutzung von Hilfsmitteln (Rollstuhl/Rollator, Sehhilfen/Blindenstock, Hörhilfen/Gebärdensprache) zur Verfügung. Die AID:A-Studie und einzelne Wellen des SOEP haben zur Stufe 2 keine Angaben.

Zur Stufe 3 (zusätzliche Erfassung von Teilhabeeinschränkungen nach Lebensbereichen und durch die Umwelt) sind in den Studien und Surveys vor allem Angaben zur sozialen Teilhabe (AID:A, „Aufwachsen und Alltagserfahrungen von Jugendlichen mit Behinderung“, DEAS, PASS, SHARE, SOEP) und zu Freizeitaktivitäten (AID:A, PASS, SOEP) vorhanden. Weitere Themen sind Erwerbstätigkeit (PASS, SHARE, SOEP) sowie Isolation, Stigma und Diskriminierung (PASS, SHARE, EU-SILC). Der Teilhabesurvey erhebt die Teilhabe in den verschiedenen Bereichen der UN-BRK. Der im RKI-Panel integrierte EHIS 4 (2025) fragte nach Teilhabe in den Bereichen Mobilität, Transport, Zugang zu öffentlichen Gebäuden, soziale Aktivitäten und Internetnutzung [18].

Die Datenquellen der amtlichen Statistik erfassen weiterhin überwiegend Angaben zu den Stufen 1 und 3 erfassen. Gleiches gilt für die Prozessdaten der DRV und der BA. Die in der Schwerbehindertenstatistik enthaltenen Angaben zur Art der Behinderung sind mit anderen Erhebungen nicht vergleichbar, da eine andere Systematik genutzt wird [19]. Darüber hinaus enthalten die Schulstatistik (Förderschwerpunkte) und die DRV-Statistik (Diagnosegruppen) Angaben zur Stufe 2. Die Angaben der Stufe 3 beziehen sich vor allem auf Erwerbstätigkeit, Einkommen und Bildung. Die BA-Statistik bezieht nur Personen mit amtlich anerkannter Schwerbehinderung oder ihnen Gleichgestellte ein und differenziert nicht nach gesundheitlichen Einschränkungen. In der DRV-Statistik sind nur die Leistungen zur Rehabilitation und Teilhabe enthalten. Deren Inanspruchnahme hängt jedoch nicht nur vom Bedarf, sondern auch von anderen Faktoren wie Zugangsmöglichkeiten oder -barrieren ab (vgl. z. B. [20]).

## Diskussion

Daten zu Gesundheit und Teilhabe von Menschen mit Beeinträchtigungen und Behinderungen in Deutschland sind mit unterschiedlichen Schwerpunkten und in unterschiedlicher Breite bzw. Tiefe in vielen Datenquellen enthalten. Die erneute Recherche nach rund 10 Jahren zeigt eine Vielzahl von großen Studien und Surveys, die für eine Gesundheits- und Teilhabeberichterstattung genutzt oder die in diese Richtung erweitert werden können.

### Verfügbare Datenquellen und ihre Nutzung

Im Vergleich zur Übersicht von [2] sind mehrere Datenquellen aus Aktualitätsgründen weggefallen: Die Studie zur Gesundheit Erwachsener in Deutschland (DEGS, 2008–2011) war ein für die Bevölkerung in Deutschland repräsentativer Befragungs- und Untersuchungssurvey des RKI, der nicht weitergeführt wurde. Ebenso wurde die Studie zur Gesundheit von Kindern und Jugendlichen in Deutschland (KiGGS) nicht weitergeführt, die letzten Daten stammen aus der Welle 2 (2014–2016). Der European Health and Social Integration Survey (EHSIS, Datenhalter: Eurostat) umfasste nur eine Erhebungswelle 2012. Eine Fortsetzung war nicht geplant; stattdessen sollte ein Modul zu Behinderung in künftige EHIS-Befragungen aufgenommen werden [2]. So werden im EHIS Welle 4 (2025) Teilhabeeinschränkungen in 5 Lebensbereichen erfragt [18]. Zur vorherigen Übersicht hinzugekommen ist der Teilhabesurvey, der als Panelstudie angelegt ist und bisher 2 Wellen umfasst (2019/2020 und 2023/2024) und als einzige der bevölkerungsweiten Studien auch qualitative Daten enthält [21, 22]. Auch wenn Gesundheit kein expliziter Schwerpunkt des Teilhabesurveys ist, hat sich damit die Datenlage insgesamt deutlich verbessert. Allerdings ergeben sich gleichzeitig Datenlücken im Bereich Kinder und Jugendliche. Das RKI entwickelt derzeit ein Konzept für ein umfassendes und kontinuierliches Kinder- und Jugendgesundheitsmonitoring (PINOKIJO-Projekt) [23]. Wie Gesundheit und Teilhabe von Minderjährigen mit Beeinträchtigungen und Behinderungen in diesem Monitoring abgebildet werden können, ist Gegenstand aktueller Recherchen. Seit Kurzem sind über das Forschungsdatenzentrum Gesundheit am Bundesinstitut für Arzneimittel und Medizinprodukte (BfArM) auch Auswertungen von Abrechnungsdaten der gesetzlichen Krankenversicherung möglich [24]. Inwieweit diese als Datenquellen zu Behinderung und Teilhabe nutzbar sind, kann Gegenstand zukünftiger Arbeiten sein.

Werden die Auswertungen der vorgestellten Datenquellen und die Nutzung für die Berichterstattung betrachtet, scheinen die Potenziale weiterhin noch nicht vollständig ausgeschöpft. Daten aus dem Teilhabesurvey, dem Mikrozensus, dem SOEP, GEDA und KiGGS werden bzw. wurden für die Teilhabeberichterstattung genutzt [8, 12, 13]. Ergebnisse des Teilhabesurveys wurden zudem in den Abschlussberichten zu den beiden Erhebungswellen veröffentlicht [21, 22]. In die Gesundheitsberichterstattung des Bundes wurden Daten der Schwerbehindertenstatistik, des Mikrozensus, der Studien GEDA/EHIS und KiGGS sowie Daten der Bildungsberichterstattung einbezogen [3, 15, 25, 26]. Im Bericht zur Gesundheit der Frauen in Deutschland wurden zudem Daten der Studie zu Lebenssituation und Belastungen von Frauen mit Behinderungen und Beeinträchtigungen [27] dargestellt. Die jährlich erscheinenden Bildungsberichte nutzen Statistiken der Kinder- und Jugendhilfe und der allgemeinbildenden Schulen zur inklusiven Bildung [28]; 2014 war das Schwerpunktthema „Menschen mit Behinderung“ [29]. Auch die wesentlichen Ergebnisse aus den Prozessdaten der DRV und der BA werden in entsprechenden Berichten zusammengefasst [30, 31]. Aus anderen Datenquellen liegen bisher nur wenige Veröffentlichungen zu Gesundheit und Teilhabe von Menschen mit Behinderungen vor (z. B. [32–34]).

### Indikatoren zur Erfassung von Behinderung und Teilhabe

Als Indikator zur Erhebung von Behinderungen wird in mehreren Befragungen die GALI-Frage genutzt [17]. Ursprünglich war dies eine einzige Frage; wegen ihrer Länge und Komplexität wird inzwischen die Erhebung mit 2 Fragen empfohlen („Are you limited because of a health problem in activities people usually do? Would you say you are … severely limited, limited but not severely, or not limited at all?“ Bei Beantwortung mit „severely limited“ oder „limited but not severely“ wird die zweite Frage gestellt: „Have you been limited for at least the past 6 months? Yes, No“) [17]. Da die GALI-Frage bereits in vielen Studien und in der amtlichen Statistik eingesetzt wird, bietet sie sich als Vergleichs- bzw. Standardindikator an. Von der Integration der GALI-Frage könnten vor allem Surveys profitieren, in denen bisher nur die amtlich anerkannte Behinderung erhoben wird. Aber auch die Information zur amtlich anerkannten Behinderung ist wichtig zu erheben, weil sie einen Vergleich mit der amtlichen Statistik ermöglicht. Ein Instrument, um funktionale Gesundheit in bevölkerungsbezogenen Gesundheitssurveys zu erheben (Stufe 2 des Stufenkonzepts), wurde von der Budapest Initiative entwickelt und umfasst Fragen zu Sehen, Hören, kognitiven Fähigkeiten, Erschöpfung und Kommunikation [35]. Ähnlich ist das aus 6 Fragen bestehende Washington Group Short Set on Functioning, das auch in einer Version für Kinder sowie in einer erweiterten Version existiert [36]. Ein Vorschlag für einen Minimalindikator für Teilhabeeinschränkungen (Stufe 3) ist, angesichts der wichtigen Rolle von sozialer Teilhabe für die Inklusion [37], das Vorhandensein sozialer Kontakte zu erfassen [2], z. B. über den Oslo 3‑Items Social Support Scale [38]. Ein umfangreicheres Instrument zur Messung von Behinderung ist der Model Disability Survey der WHO und der Weltbank, der als Stand Alone Survey genutzt oder in bestehende Befragungen integriert werden kann [39]. Mit Blick auf die Erhebungsinhalte sollte außerdem berücksichtigt werden, dass nicht nur Defizite erfragt, sondern auch Daten zu individuellen und Umweltressourcen erhoben werden [2, 8, 9].

### Studiendesign und Methodik

Die in der Übersicht enthaltenen Datenquellen sind überwiegend quantitative Erhebungen von Individualdaten. Für die Gesundheits- und Teilhabeberichterstattung sind darüber hinaus Daten relevant, die die Angebotssituation für Betroffene widerspiegeln, etwa zur Barrierefreiheit in Arztpraxen [3] oder zur Verfügbarkeit von sozialpädiatrischen Zentren (SPZ) und medizinischen Zentren für Erwachsene mit Behinderungen (MZEB). Neben quantitativen Erhebungen sind auch qualitative Erhebungen sinnvoll, um die gesundheitliche Situation, Versorgung und Teilhabe in der Wahrnehmung von Menschen mit Behinderungen detailliert beschreiben zu können. Als einzige der vorgestellten Studien enthält der Teilhabesurvey qualitative Anteile: Die quantitativen Befragungen wurden durch offene Interviews ergänzt, um die individuellen Erfahrungen der Befragten zu Themen wie Gesundheit, Partnerschaft, Familie, persönliche Sicherheit und Bildung einbeziehen zu können [22].

Weitere Limitationen der gelisteten Datenquellen sind, dass aufgrund des Studiendesigns die Teilnahme von Personen in Einrichtungen meist nicht möglich ist, Menschen mit Beeinträchtigungen und Behinderungen nicht an der Erarbeitung der Erhebungsinstrumente beteiligt sind und diese häufig nicht barrierefrei zugänglich sind (z. B. telefonische Erhebungen für Personen mit Hörbeeinträchtigungen, keine Fragebögen in leichter Sprache, Proxyinterviews nicht möglich). Zudem ist die Berücksichtigung dessen, dass Menschen mit Behinderungen eine sehr heterogene Personengruppe sind, die für die Ableitung von Handlungsbedarfen weiter differenziert werden müsste (z. B. nach der Art der Behinderung), mit den zusammengetragenen Mehrthemen-Befragungen meist nicht möglich. Ausnahme ist der Teilhabesurvey, der auch eine angepasste Erhebungsmethodik für unterschiedliche Personengruppen vorsieht [22].

## Fazit

Im Vergleich der beiden Recherchezeitpunkte 2016 und 2026 zeigt sich, dass es eine große Zahl von Datenquellen mit bevölkerungsbezogenen, regelmäßig erhobenen Indikatoren zu Beeinträchtigung, Behinderung und Teilhabe gibt. Das Potenzial für Auswertungen und Berichterstattung ist nach wie vor jedoch nicht vollständig ausgeschöpft. Mit dem Teilhabesurvey ist eine wichtige Datenquelle hinzugekommen, allerdings haben sich auch Datenlücken für das Kindes- und Jugendalter ergeben. Weiterhin besteht die Notwendigkeit, die Teilnahme an Studien barrierearm oder sogar barrierefrei zu gestalten. Daraus ergeben sich methodische Anforderungen für zukünftige Surveys. Ebenso wäre die Nutzung von Standardinstrumenten bzw. die Integration eines kurzen Instruments zur Teilhabe in die Surveys sinnvoll. Für die Gesundheitsberichterstattung könnte die Entwicklung eines Kernindikatorensets zur Gesundheit und Teilhabe von Menschen mit Beeinträchtigungen und Behinderungen nützlich sein, um auf Problemfelder und Handlungsbedarfe besser hinweisen zu können.

## Data Availability

Alle dieser Arbeit zugrunde liegenden Daten sind in diesem Artikel enthalten.
